# Spinal Curvature and Gymnastics

**Published:** 1898-06

**Authors:** Theodore Toepel

**Affiliations:** Student Atlanta Medical College; Atlanta, Ga.


### CORRESPONDENCE.

### SPINAL CURVATURE AND GYMNASTICS.

ATLANTA, GA., May 10, 1898.

It is very important that man should aim to have a symmetrically-formed body. To do justice to his life, his accomplishments and his social connection with society, it is one of the necessities to develop personal pride and character. It is therefore of the utmost importance that parents and teachers should pay close attention to the physical development of the child, thereby avoiding bodily deformities like the frequently occurring lateral curvatures. Insignificant appearances which mark the beginning of this evil are very seldom noticed by the child's parents; very often, merely by chance is the parents' attention attracted to the rapidly progressing disease. It therefore becomes the duty of every parent to watch the development of the child's body closely, and at the discovery of a suspicious sign of a possible deformity, employ medical assistance.

One of the most frequently occurring curvatures of the spinal column is scoliosis, or lateral curvature. It is, in the majority of cases, the result of faulty carriage of the trunk while walking and the wrong posture during sitting. The latter can in a great number of cases be traced back to the schoolroom, where, on account of the exorbitant demand for mental work, very little attention is paid to the physical development of the child. Statistics prove that of one thousand school children, at the ages from seven to eleven years, not less than fifty-six per cent. were afflicted with scoliosis.

Scoliosis is the result of a one-sided position of the trunk, the position being assumed involuntarily. A frequent repetition of this one-sided attitude of the trunk will cause the intervertebral cartilage to diminish on the side of the concavity and increase on the side of the convexity. The same difference will be noticed in the development of the muscles. A rotation of the vertebræ very often accompanies the lateral curvatures; this is very noticeable in the so-called high shoulders and high hips.

Symptoms like narrow chest, difficulty in breathing, indigestion, and general debility of the whole system are very often found in scoliotic people. A cure is only then possible if the disease is attended to during its first stage. I can conscientiously say that gymnastic exercise is one of the main factors for the cure of this kind of deformity. General weakness, improper nourishment, especially undeveloped muscles, weak bones and ligaments, are invariably the fundamental causes of scoliosis, and aid remarkably in the progress of this disease. It is a well known fact that gymnastic exercise is the best medicine to increase the appetite, thus stimulating and strengthening the tissues of the body.

A slight lateral curvature of the spinal column can easily be remedied by properly selected exercises, thereby improving the more or less faulty carriage. These exercises will loosen the vertebræ on the concave side; also stretching the muscles, thereby applying pressure on the convex side.

If the person that is to be treated has reached an age where it is impossible to correct the existing curvature, exercises should be prescribed that will prevent the deformity from affecting the functions of the different organs. In such an instance I would recommend the following exercise: Free-hand hanging from a bar (feet must not touch the floor), upper grip, arms extended, feet and knees together, ankles extended, chest arched and head erect; in this position practice forced respiration.

But all light deformities can be cured by gymnastic exercise. Of course it becomes more difficult if the disease is of an orthopedic nature; mechanical and surgical treatment then becomes necessary. No deformity, however, is so severe that properly applied exercise would not be of some benefit.

The first principle in this, as in any other sickness, is to prevent the development of the disease in its first stage. Parents and teachers should therefore watch with the greatest care over the physical development of the child, and, if necessary, with severity insist upon a good carriage and correct posture. In addition, practice lots of gymnastics, exercise on the horizontal bar, on the rings, etc.; also exercises with dumb-bells and wands are excellent means to avoid any kind of spinal curvature, thus giving the child the foundation for a symmetrically formed body. It is a natural and rational development of all its muscles, which will produce strength and beauty, thereby relieving the parents of worry and continued anxiety about their otherwise sickly children.

THEODORE TOEPEL,
Student Atlanta Medical College.

---

### YELLOW FEVER IN CUBA.

Dr. John Guiteras recently spoke (*Press*) of the danger necessarily arising from the occupation of Cuba by the United States troops. A great deal has been recently said of what is termed "individual precautions" against yellow fever while in an infected district, which Dr. Guiteras thinks is almost useless in time of war. He said that the disease ought not to be treated from the standpoint of the individual but from the standpoint of the mass. Possibly the most important measure to be exercised by the army would be general measures affecting the distribution of troops, the manner and place of landing, the location of distributing centers of supplies, and the selection of sites for the establishment of camps. The Island of Cuba is usually free from the disease till the middle of June, when it continues until the middle of October, being worse during the months of August, September and October. Outside of the large seaport cities the climate is most pleasant, malarial fevers and dysentery not being more prevalent than during the civil war. The interior of the island is free from the disease, while Havana and other seaport cities are the danger spots of the disease and always infected. Another important factor in the proper protection of the troops is the shipping of all food supplies from the United States. Dr. Guiteras holds to the belief that the whole island could be kept free from the disease the year round by means of proper sanitary measures, and that the island has always been a menace to the United States owing to the lack of proper precautions maintained under Spanish misrule.—*Journal American Medical Association.*

